# Molecular clues in the regulation of mini‐puberty involve neuronal DNA binding transcription factor NHLH2

**DOI:** 10.1186/s12610-021-00124-w

**Published:** 2021-03-18

**Authors:** Faruk Hadziselimovic, Gilvydas Verkauskas, Michael B. Stadler

**Affiliations:** 1 Cryptorchidism Research Institute, Children’s Day Care Center Liestal, Liestal, Switzerland; 2 Center of Children’s Surgery, Orthopedics and Traumatology, Faculty of Medicine, Vilnius University, Vilnius, Lithuania; 3 Friedrich Miescher Institute for Biomedical Research, Basel, Switzerland; 4 Swiss Institute of Bioinformatics, Basel, Switzerland

**Keywords:** NHLH2, Cryptorchidism, Infertility, Mini‐puberty, Hypothalamus‐pituitary‐testicular axis, RNA-sequencing, Single‐cell RNA-sequencing, NHLH2, cryptorchidie, séquençage d’ARN, GnRHa, mini-puberté, infertilité, l'axe hypothalamo-hypophyso-gonadique

## Abstract

Gonadotropin releasing hormone agonist (GnRHa) treatment following surgery to correct cryptorchidism restores mini-puberty via endocrinological and transcriptional effects and prevents adult infertility in most cases. Several genes are important for central hypogonadotropic hypogonadism in mammals, including many that are transcribed in both the brain and testis. However, the expression of these genes in prepubertal gonads has not been studied systematically, and little is known about the effect of hormone therapy on their testicular and neuronal expression levels. In this review, we interpret histological sections, data on hormone levels, and RNA profiling data from adult normal testes compared to pre-pubertal low infertility risk (LIR) and high infertility risk (HIR) patients randomly treated with surgery in combination with GnRHa or only surgery. We organize 31 target genes relevant for idiopathic hypogonadotropic hypogonadism and cryptorchidism into five classes depending on their expression levels in HIR versus LIR samples and their response to GnRHa treatment. Nescient-helix-loop-helix 2 (*NHLH2*) was the only gene showing a decreased mRNA level in HIR patients and an increase after GnRHa treatment. This phenomenon may reflect a broader effect of hormone treatment on gene expression in both testicular and central nervous system tissues, which could explain why the hypothalamus-pituitary-testicular axis is permanently restored by the administration of GnRHa.

## Résumé

Le traitement par l’agoniste de l’hormone de libération des gonadotrophines (GnRHa) suite à une intervention chirurgicale pour cryptorchidie rétablit la mini-puberté par des effets endocrinologiques et transcriptionnels et prévient l’infertilité adulte dans la plupart des cas. Plusieurs gènes jouent un rôle important dans l’hypogonadisme hypogonadotrope central chez les mammifères, dont certains sont transcrits à la fois dans le cerveau et les testicules. Cependant, l’expression de ces gènes dans les gonades prépubères n’a pas été étudiée systématiquement et l’effet de l’hormonothérapie sur leurs niveaux d’expression testiculaire et neuronale n’est pas connu. Dans cette revue, nous interprétons des coupes histologiques, des données sur les taux d’hormones et des données de profilage d’ARN provenant de testicules normaux adultes et des patients prépubères à faible risque d’infertilité (LIR) et à haut risque d’infertilité (HIR) traités par chirurgie en association avec la GnRHa ou seulement la chirurgie dans le cadre d’une étude randomisée. Nous organisons 31 gènes cibles pertinents pour l’hypogonadisme hypogonadotrope idiopathique et la cryptorchidie en cinq classes en fonction de leurs niveaux d’expression dans les échantillons HIR et LIR et de leur réponse au traitement par GnRHa. Nescient-helix-loop-helix 2 (NHLH2) était l’unique gène dont le niveau d’ARNm diminue chez les patients HIR par rapport aux LIR et augmente suite au traitement par GnRHa. Ce phénomène pourrait être révélateur d’un effet généralisé du traitement hormonal sur l’expression des gènes dans les tissus testiculaires et du système nerveux central. Cela pourrait expliquer pourquoi l’axe hypothalamo-hypophyso-gonadique est définitivement rétablie par l’administration de la GnRHa.

## Introduction

Re-activation of the hypothalamus-pituitary-gonadal axis occurs during the first weeks after birth and lasts approximately 6 months. During this period, serum gonadotropin and testicular testosterone, Insulin-like 3 Protein (INSL3) inhibin B, and Anti-Müllerian Hormone (AMH) levels increase [1, 2]. The differentiation of gonocytes into Ad (A dark) spermatogonia during mini-puberty is a highly critical step during germ cell development [3, 4]. This process represents the switch from a fetal pool of stem cells (gonocytes) to an adult pool of stem cells (Ad spermatogonia) that generates germ cells during a man’s entire adult life. Normal development of Ad spermatogonia depends on luteinizing hormone (LH) and testosterone [5]. However, cryptorchid infants present with different degrees of impairment of the testosterone increase during mini-puberty [6]. Patients whose mini-puberty and gonocyte differentiation are strongly impaired experience the most severe forms of infertility as adults [7]. Importantly, gonadotropin releasing hormone (GnRH) treatment permanently induces the transformation of gonocytes and undifferentiated spermatogonia into Ad spermatogonia, which rescues adult fertility in 86 % of high infertility risk (HIR) patients [8].

In this review, we interpret previously published RNA profile data obtained from testicular biopsies with a focus on genes that are implicated in idiopathic gonadotropin deficiency via genetic data [9] and additional candidate genes for isolated gonadotropin deficiency that are not yet corroborated by genetic analyses [10, 11]. Relevant samples from high and low infertility risk (HIR/LIR) patients have been described elsewhere [12–14]. RNA isolation, purification, library preparation, sequencing, data analysis, and expression analysis were reported in a previous publication [12].

Among 31 genes, only *NHLH2* shows decreased messenger ribonucleic acid (mRNA) levels in HIR versus LIR patients, whereas GnRHa treatment increases *NHLH2* mRNA. This intriguing result points to a novel role for the brain *NHLH2* transcription factor in testicular cells and raises the interesting possibility that curative hormone therapy not only influences gene expression in the testis, but in the central nervous system (CNS) as well, as *NHLH2*’s role in the brain is known to be critical for hypogonadotropic hypogonadism. We also propose possible regulatory mechanisms for multi-tissue responses to hormone treatment.

### Genes involved in central hypogonadotropic hypogonadism fall into five distinct GnRHa response classes

**Fig. 1:**
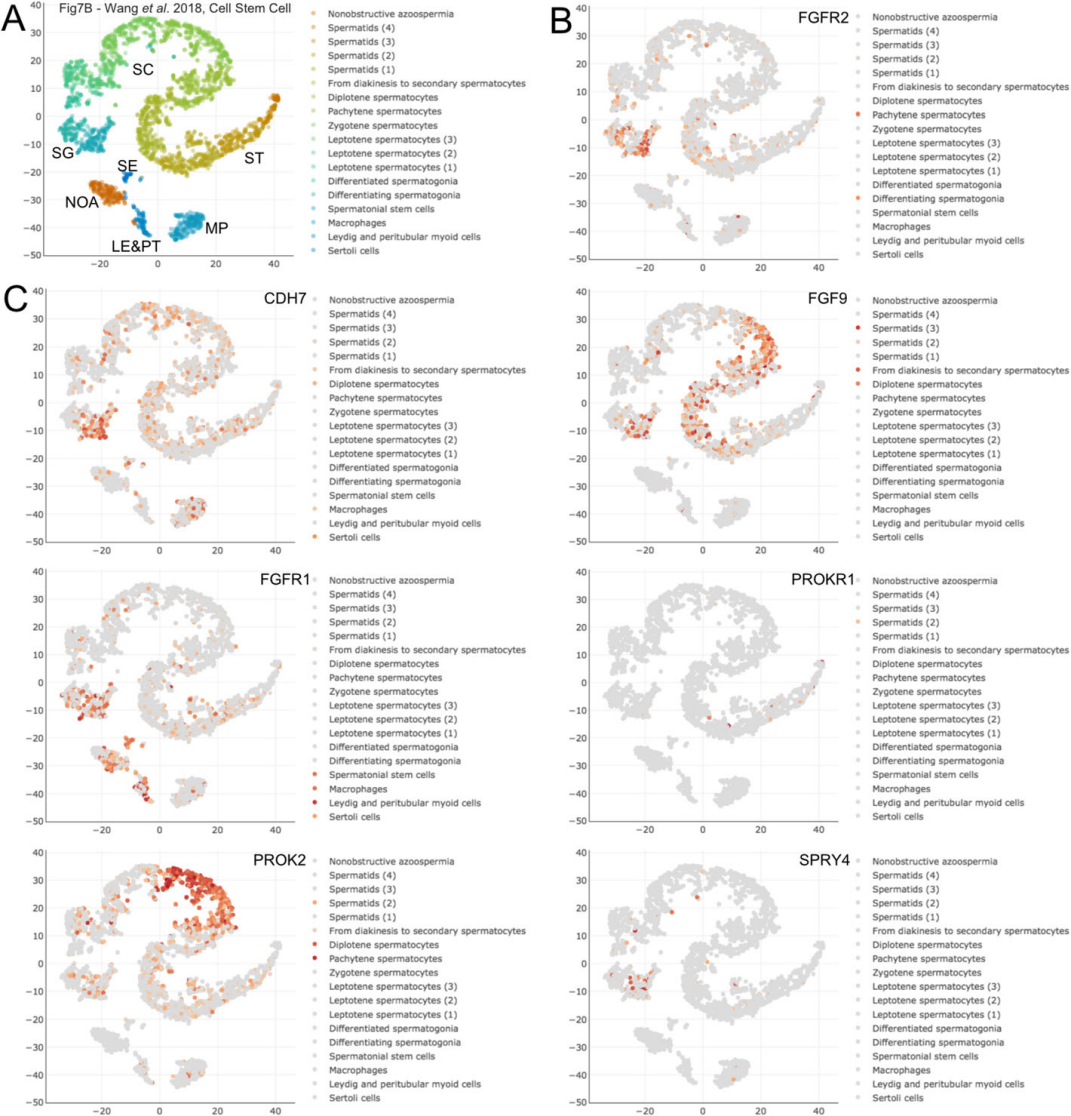
scRNA-Seq data for Class 1 genes. **a** A color-coded scatter plot shows the distribution of testicular cells. The reference is indicated at the top. The cell populations are described in the key and replicates are numbered. Broad cell types are indicated for macrophage (MP), Sertoli cell (SE), Leydig and peritubular cells (LE&PT), spermatogonia (SG), spermatocyte (SC), spermatid (ST), and non-obstructive azoospermia (NOA). **b** Expression data for Class 2 gene FGFR2. **c** Expression data for individual cells are shown in shades of red for seven Class 2 genes. The data were retrieved from the Reproductive Genomics Viewer at https://rgv.genouest.org

We interpreted our expression data from LIR/HIR and untreated/treated HIR samples for 31 genes and organized them into five classes according to their expression patterns (Table 1). Class 1 (significantly lower in HIR versus LIR and significantly lower after HIR treatment) consisted of fibroblast growth factor receptor 2 *(FGFR2*). This gene is one of four FGFRs, and the protein kinase it regulates cell division, differentiation, migration, programmed cell death, and embryogenesis [15]. Single-cell RNA sequencing (scRNA-Seq) data obtained with adult testis samples revealed that the gene’s expression peaks in a population of differentiating spermatogonia, which is consistent with its detection in biopsies from prepubertal testes that contain Ad spermatogonia (i.e., LIR; Table 1; Fig. 1A,B) [16]. The expression pattern argues against a role for this gene in idiopathic hypogonadotropic hypogonadism (IHH), which is in line with the fact that no currently known mutations associate this gene with perturbed sexual development in males [11].

**Table 1:** scRNA-Seq profile of 31 genes known to be involved in idiopathic hypogonadotropic hypogonadism (IHH) as well as genes localized downstream of Nescient-helix-loop-helix 2 (*NHL]H2)*. Expression values correspond to RPKM (Reads Per Kilobase of transcript per Million mapped reads), calculated from the model coefficients using the differential expression model described in [12]. A das]h (“-“) indicates that the gene was not detected in these samples; logFC: log2 fold-change of RNA level; FDR: false discovery rate; n.s.: not significant. HIR Ad, high infertility risk group lacking Ad spermatogonia, LIR Ad+, low infertility risk group displaying Ad spermatogonia, HIR/GnRHa, high infertility risk group, before (prior ) and after (post) GnRHa treatment

**Symbol**		HIR Ad-	LIR Ad+	logFC	FDR	HIR/GnRHa		logFC	FDR
						prior	post		
*Class 1: lower in HIR versus LIR and lower after HIR treatment*									
FGFR2	Fiboblast growth factor receptor 2	2.05	4.3	-1.25	0.0005	4.10	2.75	-0.57	0.01
*Class 2: lower in HIR versus LIR but no significant change after HIR treatment*									
CHD7	Chromodomain helicase DANN binding protein 7	3.94	6.53	-0.72	0.001	6.46	5.20	n.s.	n.s.
FGF9	Fiboblast growth factor 9	0.58	1.14	-1.06	0.001	0.81	1.20	n.s.	n.s.
FGFR1	Fiboblast growth factor receptor 1	5,26	7.40	-0.49	0.019	7.60	5.94	n.s.	n.s.
MET	MET proto-oncogene, receptor tyrosine kinase	0.40	0.98	-1.28	0.007	0.78	0.95	n.s.	n.s.
PROKR1	Prokineticin receptor 1	0.58	1.43	-1.30	0.005	1.11	0.92	n.s.	n.s.
PROK2	Prokineticin receptor 2	0.22	1.23	-2.43	0.001	0.67	0.81	n.s.	n.s.
SPRY4	Sprouty RTK signaling antagonist 4	0.57	1.18	-1.05	0.001	1.20	1.16	n.s.	n.s.
*Class 3: no significant difference between HIR and LIR and lower after HIR treatment*									
DMXL2	Dmx-like 2	9.84	12.35	n.s.	n.s.	13.31	7.58	-0.81	0.001
CXCL12	C-X-C motif chemokine ligand 12	13.57	13.81	n.s.	n.s.	16.84	9.53	-0.82	0.003
GLCE	Glucuronic acid epimerase	20.47	17.38	n.s.	n.s.	21.30	11.80	-0.85	0.0006
GNRH	Gonadotropin realeasing hormone	10.37	8.95	n.s.	n.s.	10.37	7.42	-0.48	0.035
GNRHR	Gonadotropin realeasing hormone receptor	2.15	2.26	n.s.	n.s.	2.94	1.72	-0.76	0.002
ANOS1	Anosmin 1	13.14	11.36	n.s.	n.s.	12.71	7.35	-0.79	0.0009
LEPR	Leptin receptor	3.40	3.31	n.s.	n.s.	4.16	2.56	-0.69	0.003
NDN	Necdin, MAGE family member	28.99	29.08	n.s.	n.s.	30.00	21.06	-0.77	0.001
OTUD4	OTU deubiquitinase 4	23.49	23.75	n.s.	n.s.	26.78	16.72	-0.67	0.005
TTF1	Transcription termination factor 1	7.52	7.40	n.s.	n.s.	8.76	6.20	-0.49	0.02
VEGFA	Vascular endothelial growth factor A	9.95	11.27	n.s.	n.s.	12.22	7.58	-0.68	0.005
WDR11	WD repeat domain 11	19.99	17.77	n.s.	n.s.	21.08	13.03	-0.69	0.003
*Class 4: no significant difference between HIR and LIR and higher after HIR treatment*									
CCD141	Coiled-coil domain containing 141	0.47	0.53	n.s.	n.s.	0.63	1.06	+0.74	0.003
EBF2	Early B-cell Faktor 2	0.19	0.22	n.s.	n.s.	0.24	0.84	+1.76	2.07E-05
FEZF1	Fez family zinc finger protein 1	-	-	-	-	0.14	0.52	+1.84	0.014
LEP	Leptin	0.20	0.26	n.s.	n.s.	0.02	0.80	+1.59	0.001
NHLH 1	Necient helix-loop-helix 1	-	-	-	-	0.09	0.48	+1.95	0.01
SEMA3E	Semaphorin 3E	0.66	1.08	n.s.	n.s.	0.23	0.72	+1.59	0.0001
PCK1	Phosphoenolpyruvate carboxykinse 1	-	-	-	-	0.11	0.56	+2.36	0.001
PCSK1	Protein covertase subtilising/kexin 1	0.15	0.17	n.s.	n.s.	0.15	0.71	+2,23	2.02E-05
TAC3	Tachykinin precursor 3	-	-	-	-	0.23	0.87	+1.87	0.011
TACR3	Tachykinin precursor receptor 3	-	-	-	-	0.46	0.68	+2.93	0.003
VAX1	Ventral anterior homeobox 1	-	-	-	-	0.07	0.43	+2.46	3.4E-05
*Class 5: lower in HIR versus LIR and higher after HIR treatment*									
**NHLH2**	Necient helix-loop-helix 2	**0.18**	**0.59**	**-1.65**	**0.0007**	**0.31**	**0.74**	**+1.25**	**0.01**
*NHLH2 target genes *									
NTN1	Netrin 1	0.11	0.47	-2.08	0.0005	0.28	0.51	+0.84	0.03
UNC5D	Unc-5 netrin receptor D	0.26	0.57	-1.10	0.003	0.44	0.98	+1.15	0.002
DCC	Deleted in colorectal cancer, netrin 1 receptor	0.20	0.27	n.s.	n.s.	0.26	0.62	+1.22	0.0003

Seven genes fall into Class 2 (significantly lower in HIR versus LIR but no significant change after HIR treatment). These seven genes could be epigenetically silenced and may therefore be unable to respond to GnRHa treatment. All of them, except prokineticin receptor 1 (*PROKR1*), which is detected in a small population of spermatids, and MET proto-oncogene, receptor tyrosine kinase (*MET)*, which is not detected in any testicular cells, are transcribed in adult spermatogonia or exhibit peak expression in mitotic male germ cells (Fig. 1 C). For FGF9 and PROKR1, the absence of any detectable response to hormone treatment is consistent with the lack of genetic evidence for a critical role in idiopathic hypogonadotropic hypogonadism (IHH) [11]. This indicates that FGFR1 and PROK2 may be regulated at the post-transcriptional level. However, in the cases of chromodomain helicase DANN binding protein 7 (*CHD7*), *FGFR1*, prokineticin 2 (*PROK2*), and sprouty RTK signaling antagonist 4 (*SPRY4)*, which were genetically associated with IHH the RNA-based evidence currently available for any role in curative hormone responses is inconclusive [10, 11, 17].

**Fig. 2:**
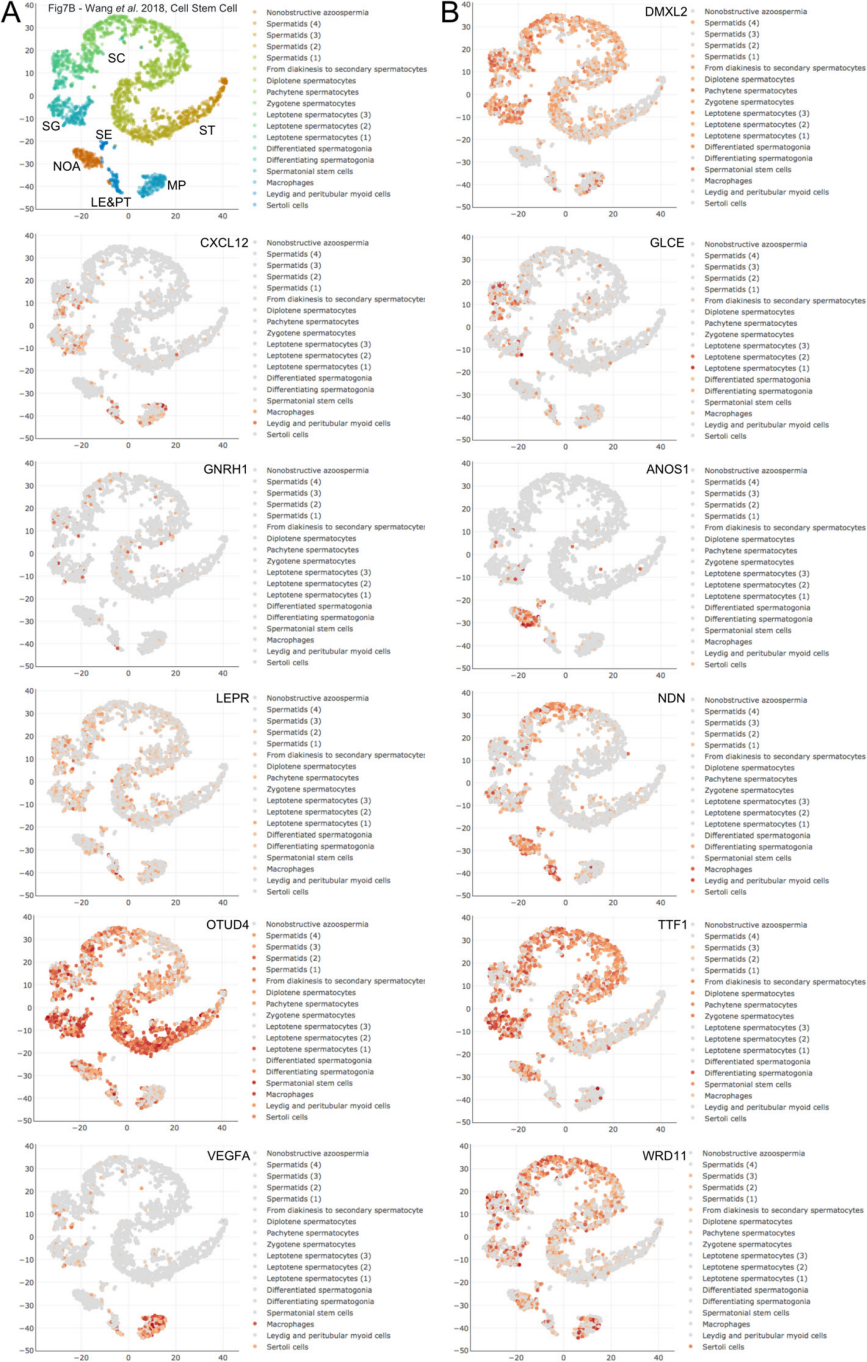
scRNA-Seq data for Class 3 genes. **a**-**b** Expression data are shown as in Fig.1

Twelve genes belong to Class 3 (no significant difference between HIR and LIR and significantly lower after HIR treatment). Dmx-like 2 (*DMXL2*), necdin (*NDN*), a MAGE family member, OTU deubiquitinase 4 (*OTUD4*), and transcription termination factor 1 (*TTF1*) exhibit strong expression in spermatogonial stem cells and some or all subsequent stages of male germ cell development (Fig. 2A, B). In contrast, leptin receptor (*LEPR*),glucuronic acid epimerase (*GLCE*), and WD repeat domain 11 (*WRD11*) are predominantly expressed in meiotic and post-meiotic germ cells, whereas anosmin 1 (*ANOS1*) and vascular endothelial growth factor A (*VEGFA*) are expressed in Sertoli cells and testicular macrophages, respectively (Fig. 2B). C-X-C motif chemokine ligand 12 (*CXCL12*) expression is detected in Leydig cells while gonadotropin releasing hormone 1 (*GNRH1*) has extremely weak, if any, expression in germ cells (Fig. 2B). It is unclear if the weaker signals for Class 3 genes in samples from treated HIR patients are relevant for the curative effect of hormone treatment.

**Fig. 3:**
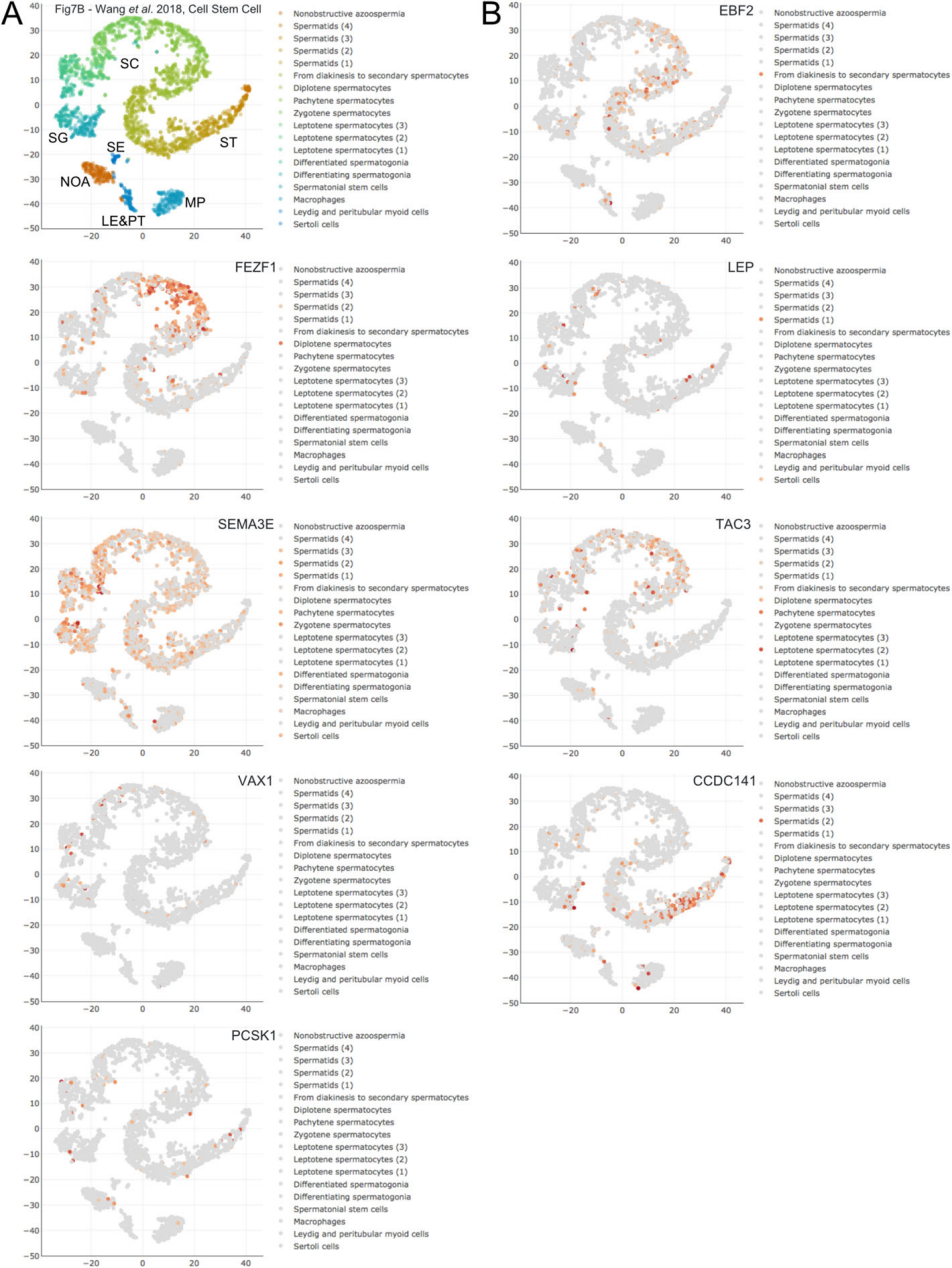
scRNA-Seq data for Class 4 genes. **a**-**b** Expression data are shown as in Fig. 1

Another 11 genes comprise Class 4 (no significant difference between HIR and LIR and significantly higher after HIR treatment). These findings indicate that GnRHa action is necessary for the central neuroendocrine control of male reproduction. For eight of these genes, the scRNA-Seq data yield interpretable results. Semaphorin 3E (*SEMA3E*) is weakly expressed in the entire male germline, whereas early B-cell Factor 2 (*EBF2*), fez family zinc finger protein 1 (*FEZF1*), and tachykinin precursor 3 (*TAC3*) mRNAs are detected in spermatocytes and coiled-coil domain containing 141 (*CCDC141*) mRNA accumulates in spermatids (Fig. 3A, B). TAC3 plays an important role in meiosis via its interaction with proteins important for this early step in spermatogenesis. This explains why TAC3 is detected in RNA profiling data of GnRHa treated patients. [18] Little, if any expression is detected for leptin (*LEP*), Protein convertase subtilisin/kexin 1 (*PCSK1*), and ventral anterior homeobox 1 (*VAX1*) (Fig. 3B).

### *NHLH2* exhibits an expression pattern indicative of a critical role in mini-puberty

**Fig. 4:**
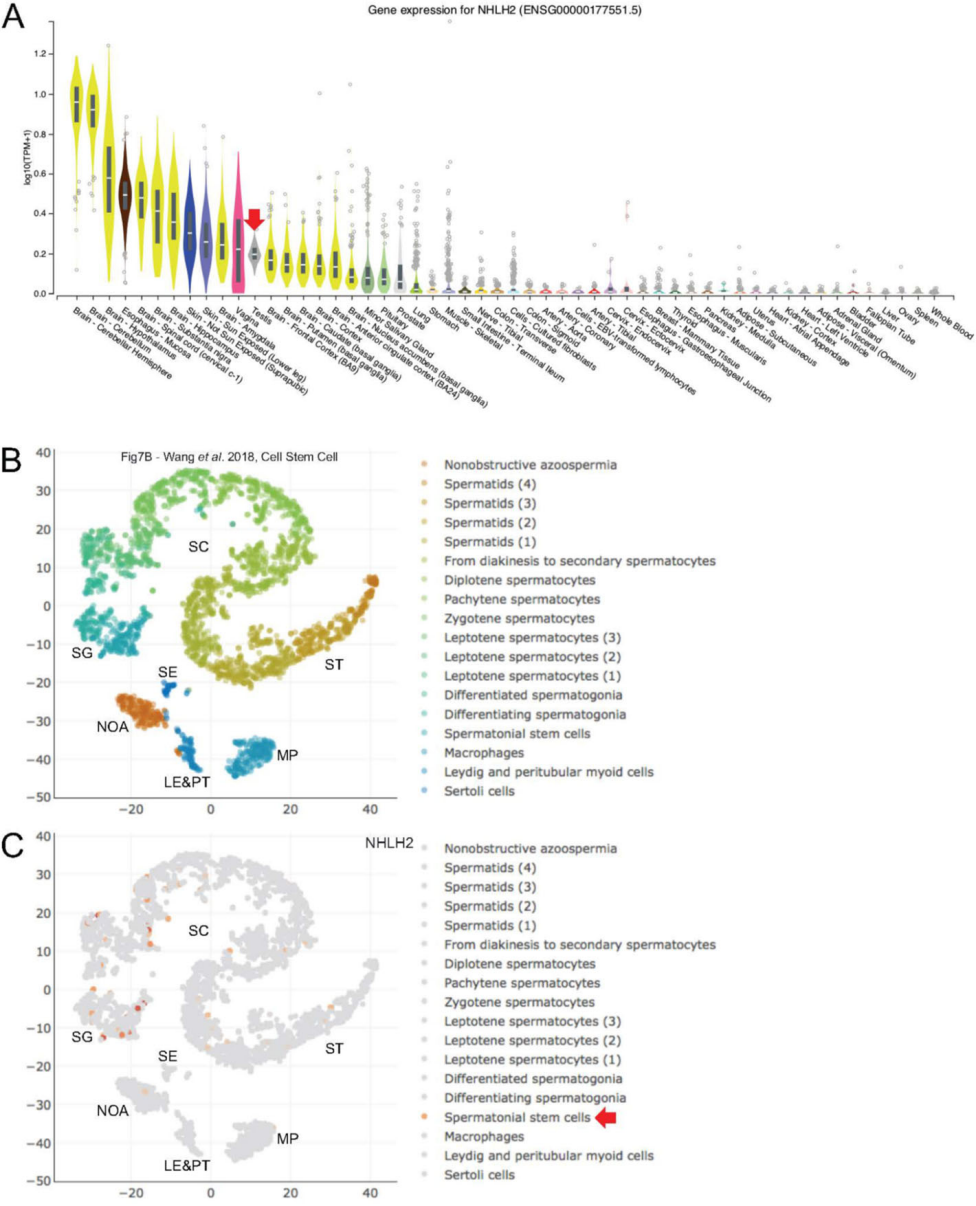
RNA profiling data for Class 5 gene *NHLH2*. **a** A color-coded violin plot shows log10 transformed RNA-Seq expression data (y-axis) for samples (x-axis) as indicated. A red arrow marks testis. The data were retrieved from the GTEX portal at www.gtexportal.org. **b**-**c** scRNA-Seq expression data are shown as in Fig. 1. A red arrow points to a key testicular cell type

*NHLH2* is the only gene in Class 5 (lower in HIR versus LIR and higher after HIR treatment) and presents an expression pattern indicative of an abnormally low mRNA level in HIR samples that is corrected by hormonal treatment (Table 1). nescient-helix-loop-helix 1 (*NHLH1*) and *NHLH2* belong to the basic-helix-loop-helix (bHLH) family of DNA binding transcription factors. Both genes are expressed in largely overlapping patterns in different areas of the central and peripheral nervous systems during the embryonic and perinatal stages [19, 20]. Male *Nhlh2*-mutant mice develop hypogonadotropic hypogonadism and are infertile, which shows that *Nhlh2* expression is critical for neuroendocrine development and maturation of the hypothalamic pituitary axis [21]. Given the gene’s known neuronal role, it is not surprising that its peak expression levels are detected in a variety of brain samples; however, a weak signal is also detected in total testis (Fig. 4A). The scRNA-Seq data from adult testes show that much of that signal is due to weak *NHLH2* expression in spermatogonial stem cells (Fig. 4B, C).

**Fig. 5:**
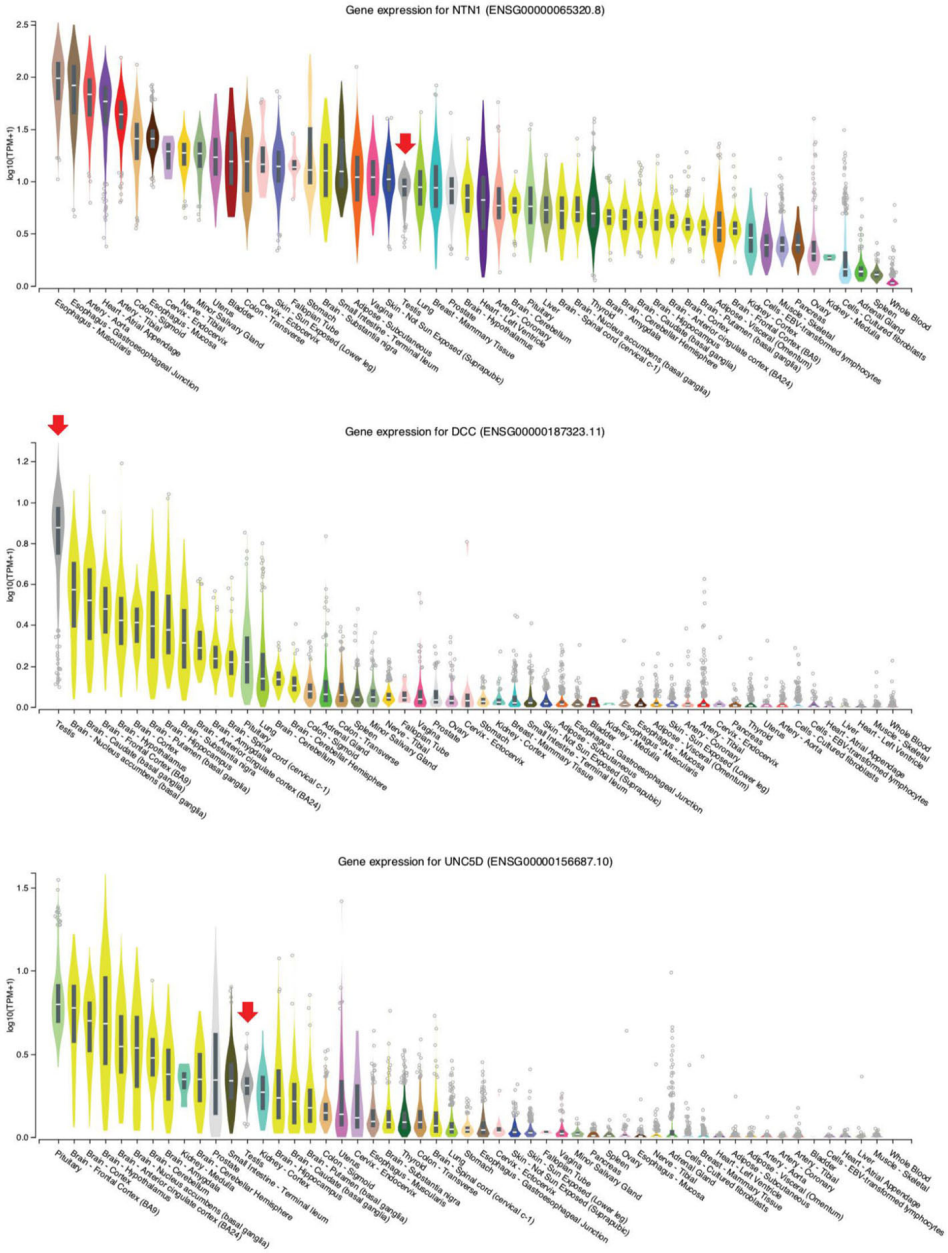
RNA-Seq data for *NHLH2 *downstream genes. Expression data are shown as in Fig. 4A

**Fig. 6:**
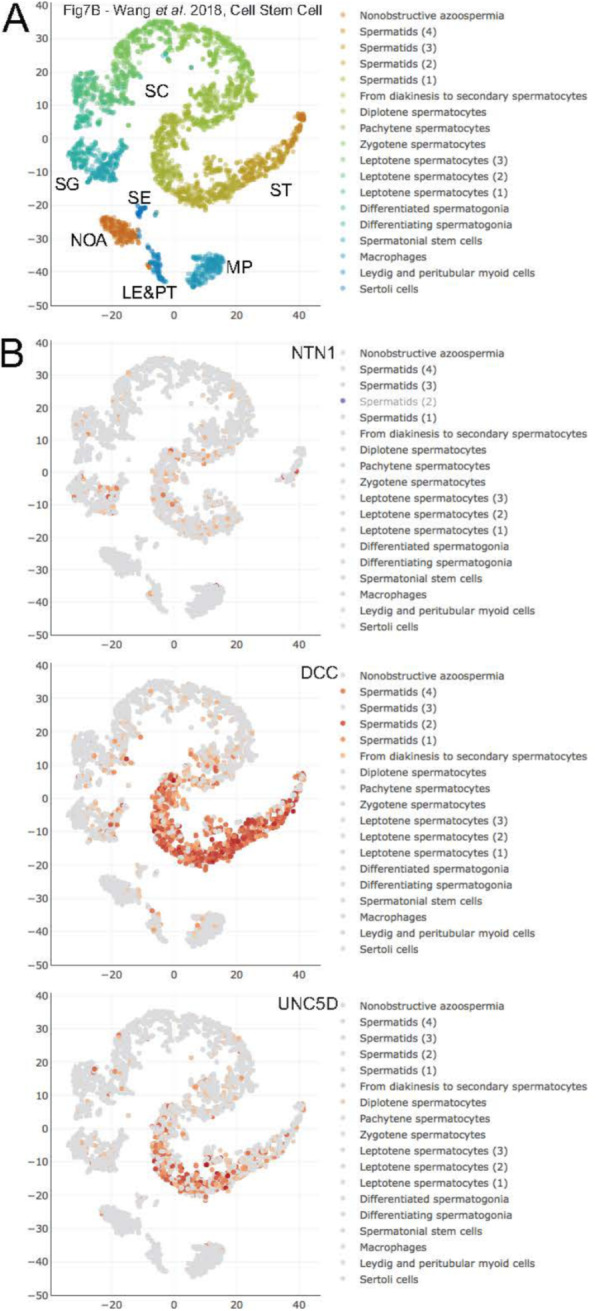
scRNA-Seq data for *NHLH2 *downstream genes. Expression data are shown as in Fig. 1

Notably, netrin 1 (*NTN1*) and unc-5 netrin receptor D (*UNC5D*) form a complex with deleted in colorectal cancer, netrin 1 receptor (*DCC*) downstream in the *NHLH2* pathway, and all three of them are expressed in both testis and brain samples (Fig. 5). More specifically, the scRNA-Seq data reveal weak (*NTN1*), medium (*UNC5D*), and strong (*DCC*) expression, predominantly in spermatids (Fig. 6A,B). Loss of fibronectin type 3 (*FN3*)-domain protein Deleted in Colorectal Cancer (*DCC*) deregulates the trajectories of a subset of vomeronasal axons that guide the migration of GnRH neurons, which leads to their abnormal localization [22]. The combined inactivation of *NHLH1* and *NHLH2* causes a complete absence of the pontine nuclei and strongly reduces the number of reticulotegmental nuclei [22]. Both genes are required for sustained expression of the netrin receptor and *DCC* in the anterior extramural migration stream [23]. Though the detailed mechanism of axon guidance is not fully understood, it is known that netrin attraction is mediated through UNC-40/DCC cell surface receptors and repulsion is mediated through UNC-5 receptors. The association of *NTN1*, which controls the guidance of commissural axons and peripheral motor axons in the CNS, with either *DCC* or *UNC5* receptors attracts or repulses axons, respectively [23]. *DCC* and its ligand *NTN1* have been shown to contain 5.2 % loss-of-function mutations in cases of central hypogonadotropic hypogonadism [24]. In humans, *NHLH*s, together with additional Lim-domain-only (LMO) cofactors, directly control transcription of the *NECDIN* gene, which is deleted in Prader–Willi syndrome with hypogonadotropic hypogonadism.

### Possible regulatory mechanisms mediating brain/testis expression of NHLH2

**Fig. 7:**
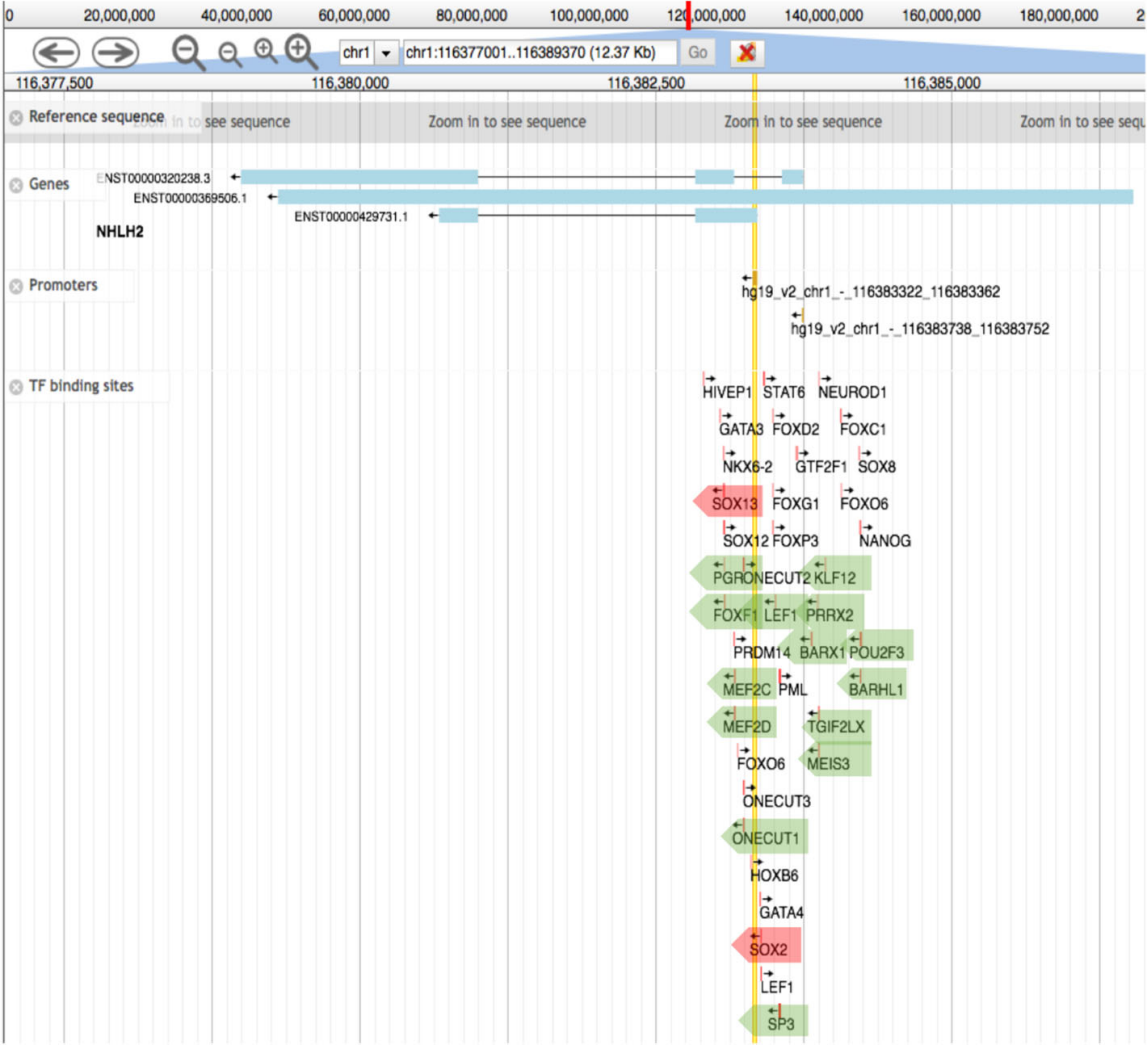
*Regulatory motif predictions for the NHLH2* promoter region. The positions and orientations of promoter motifs predicted to be involved in *NHLH2 *expression are shown. Green arrows highlight transcription factors that may be relevant. Red arrows highlight two cases that may function in the brain and testis. Genome annotation for* NHLH2* is shown at the top. The data were retrieved from SwissRegulon at https://swissregulon.unibas.ch/sr/swissregulon

**Fig. 8:**
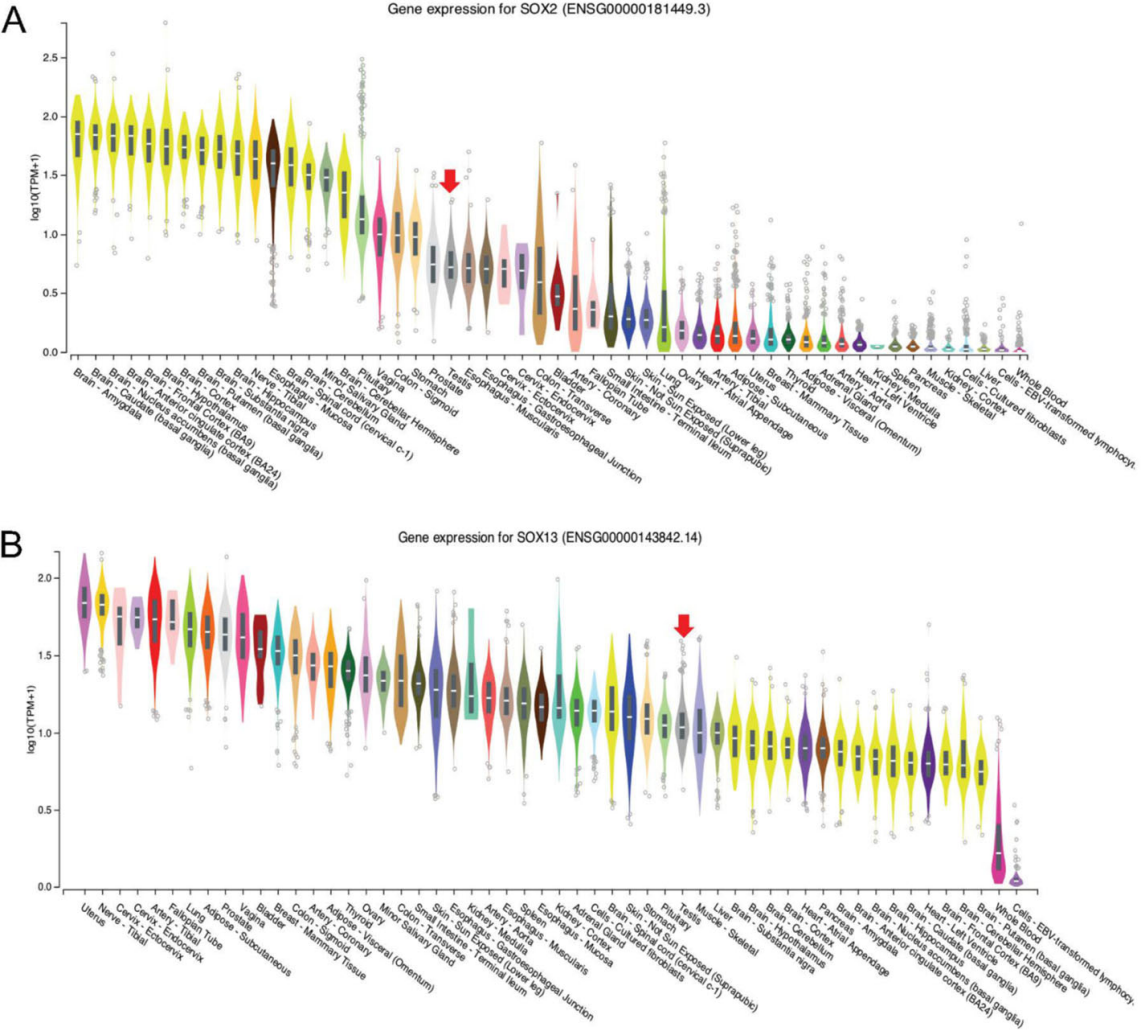
RNA-Seq expression data for* SOX* genes. **a** - **b** Violin plots for *SOX2* and *SOX13* are shown as in Fig. 4B. Red arrows indicate testis

**Fig. 9:**
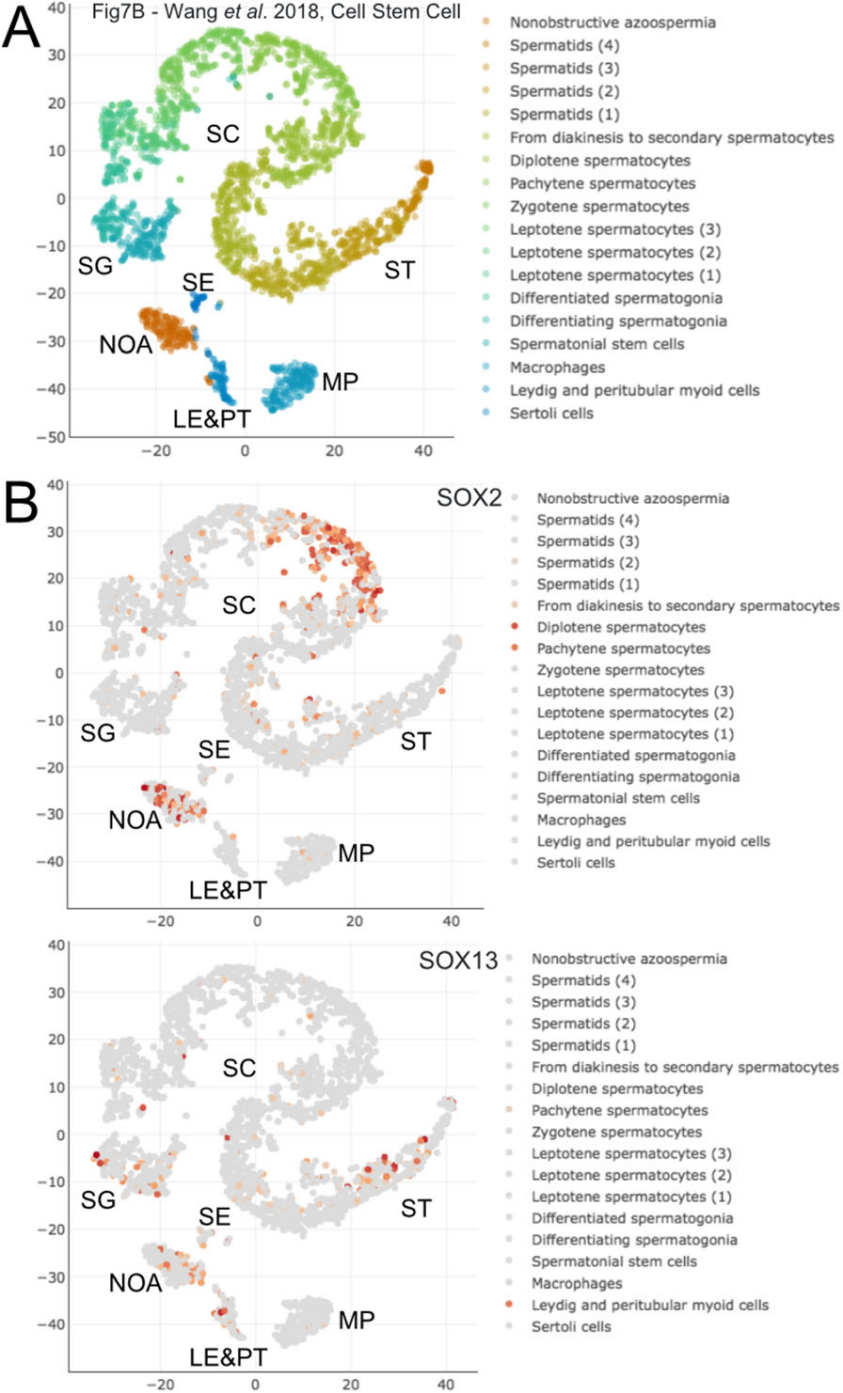
scRNA-Seq expression data for *SOX *genes. **a-b**Scatter plots are shown as in Fig. 1

The observation that *NHLH2*, which is highly relevant for IHH due to its neuronal function, appears to be induced in samples from HIR patients after hormone treatment raises the intriguing possibility that the gene’s expression is not only altered in the testis, but also the brain. It remains a matter of speculation as to what kind of regulatory mechanism may mediate an effect that simultaneously acts in distinct tissues. To explore this question, we retrieved information about *NHLH2’*s upstream promoter region. Among 16 DNA-binding regulators for which biologically functional motifs are predicted, we found SRY-box transcription factor 2 (*SOX2*) and SRY-Box Transcription Factor 13 (*SOX13*) (Fig. 7). Both are members of the Sex determining region Y (*SRY*)-box family of transcription factors, which are involved in developmental and disease processes [25]. Interestingly, both genes are expressed in the brain and testis, as one would expect (Fig. 8). Moreover, scRNA-Seq data indicate that *SOX13* is also transcribed in a sub-population of spermatogonia (and spermatids, as well as Leydig cells), whereas *SOX2* peaks in spermatocytes (Fig. 9). It is conceivable that both genes are also expressed in pre-pubertal gonocytes and Ad spermatogonia. These observations beg for further experimental evidence that may help explain how *NHLH2* is regulated in the brain and testis during mammalian post-natal development.

### A novel role for neuronal regulator *NHLH2* in testes?

RNA profiling data revealed the presence of *NHLH2* mRNA in pre-pubertal testes and in adult spermatogonia (Table 1; Fig. 4A-C). This implies that the protein is present, and possibly active, in the male germline, which raised the question of what role it may play and how it may function during the establishment of spermatogenesis. *NHLH2* physically interacts with 19 proteins, including four DNA binding transcription factors present in adult testes Hes family BHLH transcription factor 1 (*HES1*), transcription factors 3-,4-,12 (*TCF3-,4-,12*), see https://thebiogrid.org) [26]. Interestingly, *HES1* is a basic helix-loop-helix transcription factor associated with notch receptor 3 (*NOTCH3*) signaling that is expressed in prepubertal spermatogonia [27]. Moreover, TCF3 is highly specific for adult spermatogonia and was predicted to be important for male gametogenesis (see www.proteinatlas.org) [28, 29] TCF4 is expressed in germ cells and was shown to be involved in wingless-type (*WNT*) signaling, which is critical for spermatogenesis [30]. Finally, TCF12 has been genetically associated with incurable brain tumors and, therefore, may play a role in cell proliferation [31]. Moreover, *NHLH2* mRNA is bound by 75 MIRs, including several highly conserved RNAs, such as miR-93, miR130a, and miR-383, which were previously associated with spermatogenesis and fertility [32–34]. Thus, there is good evidence supporting a yet unknown role for *NHLH2* in the male germline, and further work on this question appears to be justified.

### Does GnRHa treatment affect *NHLH2* function?

Gonadotropin deficiency in cryptorchid boys with altered mini-puberty should be included in the “milder” category of IHH. Restoring mini-puberty and adult fertility is among the strongest arguments in favor of a hormone replacement therapy for the missing gonadotropin stimulus in boys with cryptorchidism and hypogonadotropic hypogonadism [8, 35]. However, only a few reports describe the outcome of gonadotropin treatment in boys with isolated central hypogonadotropic hypogonadism (CHH) within combined pituitary hormone deficiency (CPHD) during the first year of life [36–39]. Although the results from these studies show a favorable gonadotropin increase, no long-term follow-up data on these markers are available [36–39]. In contrast, two long-term studies of GnRHa-treated HIR and CHH cryptorchid boys have demonstrated that GnRHa treatment is capable of permanently correcting phenotype and rescuing fertility [8, 35]. This is most likely because GnRHa induces changes to the pathological state, which becomes apparent when analyzing LH secretion; the HIR group presents with deficient LH and testosterone secretion and, consequently, lacks Ad spermatogonia [1–4, 7]. GnRH treatment restores and normalizes LH secretion, which indicates that gonadotrophic cell differentiation occurs [8, 37, 38].

It is critical to better understand the molecular mechanisms underlying this phenomenon. One intriguing model would be that increased LH plasma levels and elevated testicular expression of *NHLH2* after treatment reflect roles of the genes in both the testis and hypothalamus during mini-puberty. The bHLH proteins encoded by *NHLH1* and *NHLH2* have been reported to fulfill important regulatory functions in the developing nervous system [40]. In mouse models, *Nhlh2* has been shown to be responsible for gonadotroph cell migration and differentiation, and that the *Nhlh2* gene has an important role in spermatogenesis [41]. Male *Nhlh2* homozygous mutant mice are microphallic, hypogonadal, and infertile with alterations in circulating gonadotropins, a defect in spermatogenesis, and a loss of instinctual male sexual behavior [21].

It has been hypothesized that mini-puberty depends more on kisspeptin-dependent GnRH-induced LH secretion than adolescent puberty [42]. However, Kiss1-metastasis suppressor (*KISS1*) gene expression was not detectable in the HIR or LIR group and had no detectable reaction to GnRHa treatment. Moreover, Nhlh2 regulates the number and distribution of GnRH neurons and the development and maturation of the adenohypophysis [35]. The combined inactivation of *Nhlh1/2* leads to a strong reduction in the reticulotegmental nuclei. Furthermore, NHLHs are required for sustained expression of the netrin receptor and cell guidance molecule DCC in the anterior extramural migration stream, and the UNC5D receptor for netrin NTN4, which promotes neuronal cell survival [22]. There are also other cases of bifunctional genes in the testis and brain. One example is *PROK2*, which is involved in central hypogonadotropic hypogonadism [3, 11]. *NHLH2* exhibits a similar pattern as *PROK2*, except that *NHLH2* responds to GnRHa treatment (Table 1).

## Conclusions

RNA profiling data from prepubertal HIR/LIR testes provide insights into the effect of curative GnRHa treatment, which permanently corrects hypothalamus-pituitary-testicular axis function, on the expression of genes important for the process. A subgroup of these genes that are known to act in neuronal tissue are also expressed in the male germline before and/or after the onset of spermatogenesis and respond to hormone treatment. Among them, we find *NHLH2*, which is involved in the functional and developmental maintenance of the hypothalamic-pituitary-gonadal axis by regulating the number and localization of GnRHa neurons, and in the development and maturation of the adenohypophysis. Critically, only *NHLH2* exhibits weaker RNA signals in HIR patients than in LIR patients and is induced by the treatment. These observations raise two intriguing possibilities. First, *NHLH2* and various other IHH genes could play novel roles specifically in male germ cells, Leydig cells, and Sertoli cells. Second, GnRHa may influence not only testicular, but also neuronal *NHLH2* expression via transcription factors that are present in both male gonads and the brain, such as *SOX2* and *SOX13*. Further research aimed at unambiguously establishing *NHLH2*’s role in controlling mini-puberty is warranted.

## Data Availability

Not applicable.

## Abbreviations

AMH: Anti-Müllerian Hormone
ANOS1: Anosmin 1
CCDC141: Coiled-coil domain containing 141
CHH: Central hypogonadotropic hypogonadism
CHD7: Chromodomain helicase DANN binding protein 7
CXCL12: C-X-C motif chemokine ligand 12
bLHL: Basic -loop-helix-loop
DCC: Deleted in colorectal cancer, netrin 1 receptor
DMXL2: Dmx-like 2
EBF2: Early B-cell Faktor 2
FEZF1: Fez family zinc finger protein 1
FGF: Fiboblast growth factor
FGFR: Fibroblast growth factor receptor
GLCE: Glucuronic acid epimerase
GnRHa: Gonadotropin releasing hormone agonist
GnRH: Gonadotropin realeasing hormone
GNRHR: Gonadotropin realeasing hormone receptor
HES1: Hes Family BHLH Transcription Factor 1
HIR: High infertility risk
IHH: Idiopathic hypogonadotropic hypogonadism
INSL3: Insulin-like 3 Protein
KISS: Kiss1-metastasis Suppressor
LH: Luteinizing Hormone
LIR: Low infertility risk
LEP: Leptin
LEPR: Leptin receptor
MET: MET proto-oncogene, receptor tyrosine kinase
NDN: Necdin, MAGE family member
NHLH2: Nescient helix-loop-helix 2
NOTCH3: Notch-receptor 3
NTN1: Netrin 1
OTUD4: OTU deubiquitinase 4
PCK1: Phosphoenolpyruvate carboxykinse 1
PCSK 1: Protein covertase subtilising/kexin 1
PROK2: Prokineticin 1
PROKR1: Prokineticin receptor 1
SEMA3E: Semaphorin 3E
SPRY4: Sprouty RTK signaling antagonist 4
SOX2: SRY-Box Transcription Factor 2
SOX13: SRY-Box Transcription Factor 13
SRY: Sex Determining Region Y
TAC3: Tachykinin precursor 3
TACR3: Tachykinin receptor 3
TCF3: Transcription factor 3
TCF4: Transrition factor 4
TCF12: Transcription factor 12
TTF1: Transcription termination factor 1
UNC5D: Unc-5 netrin receptor D
VAX1: Ventral anterior homeobox 1
VEGFA: Vascular endothelial growth factor A
WDR11: WD repeat domain 11
WNT: Wingless-type

## References

[1] Faiman C, Winter JS (1971). Sex differences in gonadotrophin concentrations in infancy. Nature.

[2] Johannsen TH, Main KM, Ljubicic ML, Jensen TK, Andersen HR, Andersen MS (2018). Sex differences in reproductive hormones during mini-puberty in infants with normal and disordered sex development. J Clin Endocrinol Metab.

[3] Hadziselimovic F (2017). On the descent of the epididymo-testicular unit, cryptorchidism, and prevention of infertility. Basic Clin Androl.

[4] Hadziselimovic F, Thommen L, Girard J, Herzog B (1986). The significance of postnatal gonadotropin surge for testicular development in normal and cryptorchid testes. J Urol.

[5] Zivkovic D, Bica DT, Hadziselimovic F (2007). Relationship between adult dark spermatogonia and secretory capacity of Leydig cells in cryptorchidism. BJU Int.

[6] Gendrel D, Job JC, Roger M (1978). Reduced post-natal rise of testosterone in plasma of cryptorchid infants. Acta Endocrinol (Copenh).

[7] Hadziselimovic F, Hoecht B (2008). Testicular histology related to fertility outcome and postpubertal hormone status in cryptorchidism. Klin Padiatr.

[8] Hadziselimovic F (2008). Successful treatment of unilateral cryptorchid boys risking infertility with LH-RH analogue. Int Braz J Urol.

[9] Stamou MI, Georgopoulos NA (2018). Kallmann syndrome: phenotype and genotype of hypogonadotropic hypogonadism. Metabolism.

[10] Miraoui H, Dwyer AA, Sykiotis GP, Plummer L, Chung W, Feng B (2013). Mutations in FGF17, IL17RD, DUSP6, SPRY4, and FLRT3 are identified in individuals with congenital hypogonadotropic hypogonadism. Am J Hum Genet.

[11] Sykiotis GP, Pitteloud N, Seminara SB, Kaiser UB, Crowley WF (2010). Deciphering genetic disease in the genomic era: the model of GnRH deficiency. Sci Transl Med.

[12] Hadziselimovic F, Gegenschatz-Schmid K, Verkauskas G, Demougin P, Bilius V (2017). Dasevicius Det al: GnRHa treatment of cryptorchid boys affects genes involved in hormonal control of the HPG axis and fertility. Sex Dev.

[13] Verkauskas G, Malcius D, Eidukaite A, Vilimas J, Dasevicius D, Bilius V (2016). Prospective study of histological and endocrine parameters of gonadal function in boys with cryptorchidism. J Pediatr Urol.

[14] Vincel B, Verkauskas G, Bilius V, Dasevicius D, Malcius D, Jones B (2018). Gonadotropin-releasing hormone agonist corrects defective mini-puberty in boys with cryptorchidism: A prospective randomized study. Biomed Res Int.

[15] Eswarakumar VP, Lax I, Schlessinger J (2005). Cellular signaling by fibroblast growth factor receptors. Cytokine Growth Factor Rev.

[16] Wang M, Liu X, Chang G, Chen Y, An G, Yan L (2018). Single-Cell RNA Sequencing Analysis Reveals Sequential Cell Fate Transition during Human Spermatogenesis. Cell Stem Cell.

[17] Barraud S, Delemer B, Poirsier-Violle C, Bouligand J, Merol JC, Grange F (2021). Congenital hypogonadotropic hypogonadism with anosmia and Gorlin features caused by a PTCH1 mutation reveals a new candidate gene for Kallmann syndrome. Neuroendocrinology.

[18] Young J, Bouligand J, Francou B, Raffin-Sanson ML, Gaillez S, Jeanpierre M (2010). TAC3 and TACR3 defects cause hypothalamic congenital hypogonadotropic hypogonadism in humans. J Clin Endocrinol Metab.

[19] Kruger M, Schafer K, Braun T (2002). The homeobox containing gene Lbx1 is required for correct dorsal-ventral patterning of the neural tube. J Neurochem.

[20] Murdoch JN, Eddleston J, Leblond-Bourget N, Stanier P, Copp AJ (1999). Sequence and expression analysis of Nhlh1: a basic helix-loop-helix gene implicated in neurogenesis. Dev Genet.

[21] Good DJ, Porter FD, Mahon KA, Parlow AF, Westphal H, Kirsch IR (1997). Hypogonadism and obesity in mice with a targeted deletion of the Nhlh2 gene. Nat Genet.

[22] Schwarting GA, Kostek C, Bless EP, Ahmad N, Tobet SA (2001). Deleted in colorectal cancer (DCC) regulates the migration of luteinizing hormone-releasing hormone neurons to the basal forebrain. J Neurosci.

[23] Schmid T, Kruger M, Braun T (2007). NSCL-1 and – 2 control the formation of precerebellar nuclei by orchestrating the migration of neuronal precursor cells. J Neurochem.

[24] Bouilly J, Messina A, Papadakis G, Cassatella D, Xu C, Acierno JS (2018). DCC/NTN1 complex mutations in patients with congenital hypogonadotropic hypogonadism impair GnRH neuron development. Hum Mol Genet.

[25] Sarkar A, Hochedlinger K (2013). The sox family of transcription factors: versatile regulators of stem and progenitor cell fate. Cell Stem Cell.

[26] Oughtred R, Stark C, Breitkreutz BJ, Rust J, Boucher L, Chang C (2019). The BioGRID interaction database: 2019 update. Nucleic Acids Res.

[27] Okada R, Fujimagari M, Koya E, Hirose Y, Sato T, Nishina Y (2017). Expression Profile of NOTCH3 in Mouse Spermatogonia. Cells Tissues Organs.

[28] Uhlen M, Fagerberg L, Hallstrom BM, Lindskog C, Oksvold P, Mardinoglu A (2015). Proteomics. Tissue-based map of the human proteome. Science.

[29] Zhu Z, Li C, Yang S, Tian R, Wang J, Yuan Q (2016). Dynamics of the Transcriptome during Human Spermatogenesis: Predicting the Potential Key Genes Regulating Male Gametes Generation. Sci Rep.

[30] Zhang H, Zhang H, Zhang Y, Ng SS, Ren F, Wang Y (2010). Dishevelled-DEP domain interacting protein (DDIP) inhibits Wnt signaling by promoting TCF4 degradation and disrupting the TCF4/beta-catenin complex. Cell Signal.

[31] Labreche K, Simeonova I, Kamoun A, Gleize V, Chubb D, Letouze E (2015). TCF12 is mutated in anaplastic oligodendroglioma. Nat Commun.

[32] Corral-Vazquez C, Salas-Huetos A, Blanco J, Vidal F, Sarrate Z, Anton E (2019). Sperm microRNA pairs: new perspectives in the search for male fertility biomarkers. Fertil Steril.

[33] Huang H, Tian H, Duan Z, Cao Y, Zhang XS, Sun F (2014). microRNA-383 impairs phosphorylation of H2AX by targeting PNUTS and inducing cell cycle arrest in testicular embryonal carcinoma cells. Cell Signal.

[34] Li C, Yang B, Pan P, Ma Q, Wu Y, Zhang Z (2018). MicroRNA-130a inhibits spermatogenesis by directly targeting androgen receptor in mouse Sertoli cells. Mol Reprod Dev.

[35] Hadziselimovic F, Herzog B (1997). Treatment with a luteinizing hormone-releasing hormone analogue after successful orchiopexy markedly improves the chance of fertility later in life. J Urol.

[36] Bougneres P, Francois M, Pantalone L, Rodrigue D, Bouvattier C, Demesteere E (2008). Effects of an early postnatal treatment of hypogonadotropic hypogonadism with a continuous subcutaneous infusion of recombinant follicle-stimulating hormone and luteinizing hormone. J Clin Endocrinol Metab.

[37] Lambert AS, Bougneres P (2016). Growth and descent of the testes in infants with hypogonadotropic hypogonadism receiving subcutaneous gonadotropin infusion. Int J Pediatr Endocrinol.

[38] Papadimitriou DT, Chrysis D, Nyktari G, Zoupanos G, Liakou E, Papadimitriou A, Mastorakos G (2019). Replacement of male mini-puberty. J Endocr Soc.

[39] Sarfati J, Dode C, Young J (2010). Kallmann syndrome caused by mutations in the PROK2 and PROKR2 genes: pathophysiology and genotype-phenotype correlations. Front Horm Res.

[40] Brown L, Espinosa R, Le Beau MM, Siciliano MJ, Baer R (1992). HEN1 and HEN2: a subgroup of basic helix-loop-helix genes that are coexpressed in a human neuroblastoma. Proc Natl Acad Sci U S A.

[41] Cogliati T, Delgado-Romero P, Norwitz ER, Guduric-Fuchs J, Kaiser UB, Wray S (2007). Pubertal impairment in Nhlh2 null mice is associated with hypothalamic and pituitary deficiencies. Mol Endocrinol.

[42] Shahab M, Lippincott M, Chan YM, Davies A, Merino PM, Plummer L (2018). Discordance in the dependence on Kisspeptin signaling in mini puberty vs adolescent puberty: human genetic evidence. J Clin Endocrinol Metab.

